# A Case of Wernicke's Encephalopathy With Objective Evaluation of Pupil Findings Using a Pupillometer

**DOI:** 10.7759/cureus.77034

**Published:** 2025-01-06

**Authors:** Shunsuke Kimura, Kota Mori, Tadataka Mizoguchi, Takahiro Kuwashiro, Hiroshi Sugimori, Yasushi Okada

**Affiliations:** 1 Department of Cerebrovascular Medicine and Neurology, Clinical Research Institute, National Hospital Organization Kyushu Medical Center, Fukuoka, JPN; 2 Department of Cerebrovascular Medicine and Neurology, National Hospital Organization Kyushu Medical Center, Fukuoka, JPN

**Keywords:** miosis, pupillometer, thiamin, vitamin b1 deficiency, wernicke encephalopathy

## Abstract

Wernicke encephalopathy is a nutritionally disabling encephalopathy resulting from thiamine deficiency. The typical triad of symptoms in Wernicke's encephalopathy includes impaired consciousness, ocular motility disturbances, and ataxic gait. Although constricted pupils and diminished light reflexes are recognized features of Wernicke's encephalopathy, few reports have specifically focused on these findings. Here, we highlight the pupil diameter and light reflex abnormalities observed in this case. A 73-year-old man was admitted to our hospital because of dizziness and then referred to our department four days later because of impaired consciousness. He presented with constricted pupils, diminished light reflexes, restricted eye movements, nystagmus, muscle weakness, and ataxia in the trunk and limbs. Magnetic resonance imaging revealed lesions in the cerebellar vermis, brain stem, and bilateral medial thalamus, leading to a diagnosis of Wernicke's encephalopathy. Following thiamine supplementation, his symptoms and magnetic resonance imaging findings improved. His pupil diameter and light reflexes, as assessed with a pupillometer, also improved over time. Tracking pupillary findings using the pupillometer may be useful in determining treatment efficacy in Wernicke's encephalopathy and is reported here.

## Introduction

Wernicke's encephalopathy (WE), first described by Carl Wernicke in 1881, is a nutritionally disabling encephalopathy resulting from thiamine deficiency [1]. The exact prevalence and incidence of WE are unknown; however, necropsy studies in adults have reported incidence rates ranging from 0.5% to 2.8%. The typical triad of symptoms includes impaired consciousness, ocular motility disturbances, and ataxic gait [2]. However, only 16% to 38% of patients have all three symptoms, and the diagnosis is often delayed in clinical practice [3,4]. Early diagnosis and treatment are crucial because initiating treatment at an early stage is more effective, reducing the likelihood of sequelae. Hence, internists must be aware of the diverse symptoms of WE. One of the common symptoms of WE is miosis [5], and reports have also indicated that the light reflex may be weakened [5-7].

Evaluation of the pupil diameter and light reflex often lacks objectivity. However, these indices can be easily, quantitatively, and objectively assessed with the use of an automatic pupillometer. This case report aims to describe a patient with WE who presented with miosis and diminished light reflexes, which were quantitatively evaluated and monitored using an automated pupillometer.

## Case presentation

This case involved a 73-year-old man who had undergone a total gastrectomy for gastric cancer 26 years earlier. He had not consumed alcohol since the gastrectomy. Before his current admission to our hospital, he had experienced dizziness for seven days and had visited the emergency room five days before admission. A computed tomography (CT) scan and magnetic resonance imaging (MRI) scan of the head showed no abnormalities, and he was discharged. However, he vomited several times and was able to ingest only a small amount of porridge. Four days before admission, he consulted an otolaryngologist and was prescribed mecobalamin. Three days before admission, he returned to the emergency room because of vomiting but was discharged when his dizziness improved upon arrival at our hospital. He was finally admitted to our hospital after visiting the otolaryngologist, who had observed nystagmus.

**Figure 1:**
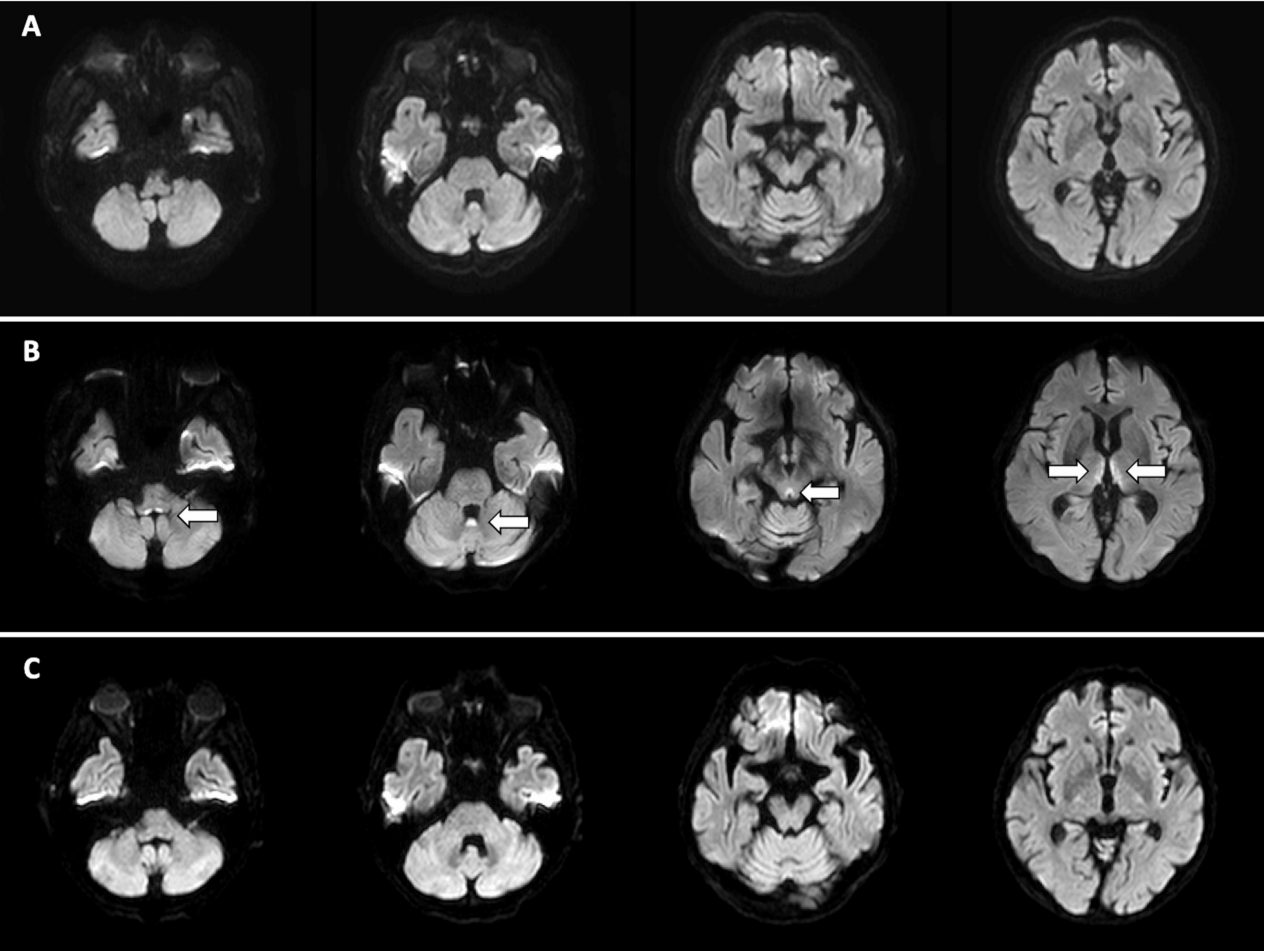
DWI findings over time. (A) DWI on admission revealed no abnormalities. (B) DWI on day 4 revealed hyperintense lesions in the bilateral dorsomedial thalamus, mammillary bodies, periaqueductal gray matter, dorsal medulla, and cerebellar vermis (arrows). (C) DWI showed that all abnormalities had disappeared by day 17. DWI, diffusion-weighted imaging

At admission, the patient’s clinical characteristics and vital signs were as follows: height, 167 cm; weight, 52.8 kg; body mass index, 18.9 kg/m²; blood pressure, 131/69 mmHg; heart rate, 81 beats per minute; body temperature, 36.8°C; respiratory rate, 18 breaths per minute; and oxygen saturation, 98% on room air. He had a Glasgow Coma Scale score of E4V5M6. There were no abnormalities in his heart or breath sounds. He exhibited upward and bilateral horizontal gaze-evoked nystagmus. Neither cranial nerve abnormalities nor gross weakness of the extremities was observed. Laboratory tests showed no significant abnormalities. A head MRI scan also showed no abnormalities (Figure 1A). The patient was admitted to our otorhinolaryngology department for follow-up. After admission, he had poor food intake and was given glucose-containing infusions that did not contain vitamin B1.

Four days later, the patient was transferred from the Otorhinolaryngology Department to the Neurology Department because he had developed impaired consciousness and restricted eye movements. His consciousness level was E3V4M6 on the Glasgow Coma Scale. On examination, he exhibited bilateral lateral gaze palsy with normal vertical gaze, and his convergence reflex was absent. His bilateral pupils showed constrictions, and the light reflex was diminished. Persistent stuttering was noted. His visual acuity and visual fields were normal, and the remaining cranial nerves were intact. Muscle weakness and ataxia of the trunk and extremities were observed, and he was unable to maintain a sitting position by himself. His perception of touch and pin-prick sensation over the limbs was normal. The deep tendon reflexes were absent, but no pathological reflexes were observed. The laboratory test results at the initial presentation are shown in Table 1. They revealed elevated concentrations of blood urea nitrogen, creatinine, and glucose, as well as mild anemia. Slightly low concentrations of serum sodium and chloride were also observed. Miller Fisher syndrome and neuromyelitis optica spectrum disorder were considered differential diagnoses, but the anti-GQ1b antibody, anti-GM1 antibody, and anti-AQP4 antibody were absent. A cerebrospinal fluid (CSF) examination revealed a pressure of 11 cmH2O. The CSF cell count and protein level were not increased. Bacterial culture, May-Grünwald Giemsa staining, and qualitative polymerase chain reaction for mycobacteria in the CSF were negative. Repeat MRI of the head revealed high signal intensity on diffusion-weighted imaging (DWI) in the bilateral dorsomedial thalamus, mammillary bodies, periaqueductal gray matter, dorsal medulla, and cerebellar vermis (Figure 1B), and these typical imaging findings led to the diagnosis of WE. Because the patient had no history of alcohol consumption, his previous gastrectomy was assumed to be the cause of his thiamine deficiency.

**Table 1:** Laboratory tests. Laboratory tests during the patient’s initial visit to our department revealed elevated concentrations of blood urea nitrogen, creatinine, and glucose, along with mild anemia. Slightly low concentrations of serum sodium and chloride were also observed. Nineteen days after admission, a markedly low level of vitamin B1 was identified. WBC, white blood cells; RBC, red blood cells; HGB, hemoglobin; HCT, hematocrit; MCV, mean corpuscular volume; MCH, mean corpuscular hemoglobin; MCHC, mean corpuscular hemoglobin concentration; PLT, platelet

Test	Results/Units	Normal range
WBC	7.10 x 10^3^/µL	3.3-8.6
RBC	3.47 x 10^3^/µL	4.35-5.55
HGB	10.6 g/dL	13.7-16.8
HCT	31.2	40.7-50.1
MCV	90 fL	83.6-98.2
MCH	30.5 pg	27.5-33.2
MCHC	39 g/dL	31.7-35.3
PLT	23.6 x 10^6^/µL	158-348
Sodium	133 mmol/L	138-145
Potassium	4.2 mmol/L	3.6-4.8
Chloride	98 mmol/L	101-108
Urea Nitrogen	53 mg/dL	8-20
Creatinine	1.13 mg/dL	0.65-1.07
Glucose	230 mg/dL	73-109
Vitamin B1	8 ng/mL	24-66
Vitamin B12	>1500 pg/mL	180-914

He immediately received intravenous thiamine (1,500 mg/day for three days, followed by 250 mg/day for three days), then continued treatment with oral thiamine (100 mg/day). Chlorpromazine therapy was initiated for stuttering. Nerve conduction studies revealed electrodiagnostic evidence of moderate motor polyneuropathy with predominantly axonal features. An electroencephalogram showed no seizure activity, but diffuse slow waves consistent with metabolic encephalopathy were present. The eye movement disorders resolved seven days after admission, and the stuttering disappeared after 14 days. Although the patient’s loss of consciousness was temporarily exacerbated by the side effects of chlorpromazine, he regained consciousness and clarity after 15 days. The muscle weakness and ataxia in the extremities gradually improved but remained, and the patient required assistance to stand up. Nineteen days after admission, a blood test during the patient’s initial visit to our department revealed a markedly low level of vitamin B1, confirming the diagnosis of WE. His vitamin B12 level was high because of the mecobalamin therapy that had been prescribed by the previous physician. Vomiting was observed on this same day, and the patient received temporary treatment with intravenous thiamine from 19 to 23 days after admission.

At 17 days after admission, the previously observed high signal intensity that had been noted on DWI in the bilateral dorsomedial thalamus, mammillary bodies, periaqueductal gray matter, dorsal medulla, and cerebellar vermis had disappeared (Figure 1C).

The patient’s left pupil was examined at 4, 9, and 17 days after admission using a pupillometer (NPi-200; NeurOptics, Irvine, CA). The measurement timings were selected based on the initial assessment, post-thiamine treatment, and MRI evaluation schedule. The following data were recorded: maximum pupil size, minimum pupil size, pupil change (%) [(maximum pupil size − minimum pupil size)/maximum pupil size], average constriction velocity, maximum constriction velocity, latency from light stimulation to contraction, and average dilation velocity. The operator centered the pupil by pressing the button corresponding to the left eye and then released the button, generating a light burst that elicited the light reflex. The pupillometer then recorded the pupillary responses, displaying the results on its screen. The findings are shown in Table 2, and data collected from 10 volunteers with no history of eye disease (median age 63 years; 7 women, 3 men) are also provided for reference. The maximum pupil size at 4, 9, and 17 days after admission was 2.32, 3.07, and 2.95 mm, respectively. The minimum pupil size was 1.61, 1.95, and 1.77 mm, respectively. The pupil change increased over time by 31%, 36%, and 40%. The average constriction velocity was 1.18, 2.44, and 2.29 mm/s; the maximum constriction velocity was 2.03, 3.44, and 3.44 mm/s; and the average dilation velocity was 0.75, 1.09, and 1.12 mm/s at each respective time point. The latency from light stimulation to contraction shortened over time by 0.33, 0.27, and 0.23 s, respectively. The improvement in pupil constriction and light reflexes generally corresponded with the improvement in the head MRI findings.

**Table 2:** Pupillometer-recorded pupil findings over time. *Control group: Data collected from 10 volunteers with no history of eye disease are provided for reference. These data are presented as median (interquartile range). Max, maximum pupil size; Min, minimum pupil size; CH, pupil change [(maximum pupil size − minimum pupil size)/maximum pupil size]; CV, average constriction velocity; MCV, maximum constriction velocity; LAT, latency from light stimulation to contraction; DV, average dilation velocity

Parameters	Day 4	Day 9	Day 17	Control group* (*n* = 10)
Max (mm)	2.32	3.07	2.95	3.46 (3.32-3.87)
Min (mm)	1.61	1.95	1.77	2.55 (2.38-2.73)
CH (%)	31	36	40	29 (24-31)
CV (mm/s)	1.18	2.44	2.29	1.91 (1.67-2.28)
MCV (mm/s)	2.03	3.44	3.44	2.86 (2.38-3.28)
LAT (s)	0.33	0.27	0.23	0.27 (0.27-0.29)
DV (mm/s)	0.75	1.09	1.12	1.20 (0.89-1.91)

The Mini-Mental State Examination was administered at 16, 30, and 37 days for neuropsychological testing. The patient improved to age-appropriate levels, scoring 22, 23, and 25, respectively. He was transferred to a convalescent hospital after 39 days primarily because of muscle weakness caused by polyneuropathy, making it difficult for him to be discharged home.

## Discussion

To the best of our knowledge, almost no reports focus specifically on the ocular findings of WE. This is the first report of WE in which a pupillometer was used to assess pupil diameter and diminished light reflexes and track their improvement. Because WE can cause serious sequelae if diagnosed too late, it is crucial to obtain a thorough history and recognize and evaluate its diverse symptoms.

One of the key points enabling diagnosis in this case is that the patient had undergone surgery for gastric cancer. The thiamine transporter is involved in the absorption of vitamin B1 and is expressed throughout the gastrointestinal tract. However, it is most abundant in the liver, stomach, duodenum, jejunum, colon, cecum, rectum, and ileum, in that order [8]. Gastric acid also stabilizes vitamin B1, which is consumed during glucose metabolism [9]. Our patient was considered to have chronic vitamin B1 deficiency due to his previous gastrectomy. Dizziness and anorexia appeared at first, followed by nystagmus. The patient had poor food intake after admission, but he was receiving glucose-containing infusions without vitamin B1 supplementation. These infusions may have triggered the development of WE.

Another key diagnostic factor was the patient’s variety of ocular manifestations. One common symptom of WE is constricted pupils [5]. The sympathetic nerves of the pupillary system originate in the hypothalamus and descend in the brain stem to synapse with prejunctional neurons from the eighth cervical vertebra to the second thoracic vertebra. From there, preganglionic fibers travel to the maxillary ganglion, and then postganglionic fibers continue along the internal carotid artery and ophthalmic artery into the brain, where they distribute to the pupillary dilator muscle of the eye [10]. In patients with WE, MRI shows abnormal signals around the middle cerebral aqueduct, periventricular third ventricle, lid plate, mamillary body, and medial thalamus [5,11,12], and these disorders are thought to cause constriction of the pupils.

WE is also known to cause diminished light reflexes [5-7]. Thiamine deficiency in monkeys reportedly causes gliosis and loss of the oculomotor nucleus [13]. WE is characterized by abnormal MRI signals around the midbrain aqueduct, including the oculomotor nucleus and Edinger-Westphal nucleus [5,11,12], which are central to the light reflex. These adverse effects on the oculomotor and Edinger-Westphal nuclei are thought to weaken the light reflex in patients with WE. Thus, the lesions responsible for the constricted pupils and attenuated light reflexes are the sites of characteristic abnormalities on MRI. In the present case, the recovery of the pupil diameter and light reflex velocity, as assessed by a pupillometer, was generally consistent with the normalization of the MRI findings.

The effectiveness of treatment for WE is primarily judged by the improvement in the patient’s symptoms. However, evaluation of symptoms, particularly pupillary findings, often lacks objectivity. A pupillometer provides objective and quantitative information about the pupil size and pupillary light reflex [14]. In patients with WE who exhibit miosis and attenuated light reflexes, examination of the pupil diameter and light reflexes with a pupillometer may be useful in determining the effectiveness of treatment. Although its use in assessing the efficacy of treatment is limited to patients with pupillary abnormalities, evaluation of pupil findings during the initial visit may also be useful in determining severity. More cases need to be accumulated for further evaluation.

## Conclusions

We report a case of WE in which the improvement of miosis and light reflexes was tracked using a pupillometer. Lesions responsible for pupillary constriction and diminished light reflexes are characteristic abnormalities observed on MRI in WE. Despite its widespread recognition, early diagnosis remains challenging. Attention to pupillary findings and light reflexes can facilitate timely diagnosis, and tracking pupil diameter and light reflexes with a pupillometer may provide valuable insights into treatment efficacy.
